# A Hand-Held Platform for Boar Sperm Viability Diagnosis Based on Smartphone

**DOI:** 10.3390/bios13110978

**Published:** 2023-11-08

**Authors:** Yunhong Zheng, Hang Yin, Chengxian Zhou, Wei Zhou, Zhijie Huan, Weicheng Ma

**Affiliations:** 1 School of Electrical Engineering and Automation, Xiamen University of Technology, Xiamen 361024, China; yhzheng666@163.com (Y.Z.);; 2 Xiamen Key Laboratory of Frontier Electric Power Equipment and Intelligent Control, Xiamen 361024, China; 3 School of Electrical and Information Engineering, Jiangsu University, Zhenjiang 212013, China

**Keywords:** sperm motility, image processing, miniature microscope, microfluidic chip, smartphone

## Abstract

The swine fever virus seriously affects pork production, and to improve pork production, pig breeding efficiency needs to be improved, and the detection of boar sperm activity is an important part of the pig breeding process. Traditional laboratory testing methods rely on bulky testing equipment, such as phase-contrast microscopes, high-speed cameras, and computers, which limit the testing scenarios. To solve the above problems, in this paper, a microfluidic chip was designed to simulate sperm in the oviduct with a channel thickness of 20 um, which can only accommodate sperm for two-dimensional movement. A miniature microscope system which can be used in combination with a smartphone is designed that is only the size of the palm of the hand and has a magnification of about 38 times. An intelligent diagnostic app was developed using Java language, which can automatically identify and track boar sperm with a recognition rate of 96.08% and an average tracking rate of 86%. The results show that the proposed smartphone-based hand-held platform can effectively replace the traditional microscope compound computer to diagnose sperm activity. In contrast, the platform is smaller, easier to use and is not limited by the usage scenarios.

## 1. Introduction

Pork is one of the major meat supplies in the world, and China is a major producer and consumer of pork [1]. According to FAOSTA, as of 2020, China ranks first in pork production with 42.8 million tons per year, more than the combined pork production of the remaining top 10 producing countries. However, in the past few years, the swine fever virus has been rampant, leading to a decline in pork production and an increase in pork prices [2]. To ensure production, the activity of boar sperm is an important indicator for breeding [3].

For the detection of sperm activity, the more widely used is the use of a computer-based system called computer-assisted sperm analysis (CASA), which is a combined software and hardware system, which has been developed and refined in the last decades [4,5,6]. CASA can obtain sperm movement trajectories and calculate parameters such as the velocity of curve line (VCL), velocity of straight line (VSL), and velocity of average path (VAP) [7]. However, CASA requires expensive hardware costs (e.g., phase contrast microscopes, high-speed cameras, computer processing systems) [8], so it is generally used only in large institutions or hospitals for human male sperm testing, and it is difficult to spread to small and medium-sized breeding institutions for boar sperm testing. Currently, boar sperm testing is widely performed using the traditional method of manual observation with a microscope, which relies on the experience of the inspector and is more subjective, without detailed parameter analysis, and the results analyzed by different inspectors sometimes vary greatly [9]. In addition, the traditional microscopes and computers used are large, the operation process is complicated, and the use scenarios are limited, so instant detection cannot be achieved. At the same time, sperm survival time in vitro is less than two hours [10], and the longer the sperm is exposed for, the lower its activity [11,12].

Microfluidics is a new technology that deals with microscale fluid flow and is a method that can be used to simulate the in vivo environment of microdevices [13,14,15]. Microfluidics can control the surrounding chemical and physical environment, simulating the in vivo environment and avoiding the risk of distorting the behavior of sperm cells when they are removed from their natural environment. Currently, microfluidics has been widely used for sperm separation [16], sperm sorting [17], and in vitro fertilization (IVF) [18]. In recent years, with the improvement in smartphone performance and the rapid development of microfluidics, the use of microfluidic chip composite smartphones to study microbial images has become a hot topic. For example, Shreya Deshmukh et al. used a microfluidic integrated cell phone imaging system to validate the testing of sperm in sexual assault samples to greatly accelerate the sample screening process for forensic investigations [19]. Zheng LY et al. developed a novel biosensor combined with a smartphone to detect changes in concentration based on images of *E. coli* O157:H7 [20]. Ulep, TH et al. designed a smartphone-based custom fluorescence microscope with a two-layer paper microfluidic chip and developed software to process and automatically count cancer cell images to instantly quantify cancer cell concentrations [21]. However, the improved portability compared to microscopes and computers sacrifices some of the performance, leading to a lack of clarity of the acquired images and a decrease in image processing power, thus requiring more applicable detection algorithms that can cope with the above situation.

In this paper, we design a hand-held platform for boar sperm viability diagnosis based on a smartphone, including a microscope device for use with a smartphone, which is the size of the palm of the hand, and a microfluidic chip with a channel thickness of 20 microns that can simulate the movement of sperm in the oviduct. Finally, we developed an app using a Gaussian mixture model, a Kalman filter, and a Hungarian algorithm capable of diagnosing sperm activity. With this hand-held system, we were able to achieve a high detection accuracy of sperm morphology and to track the movement of each sperm for viability diagnosis.

## 2. Materials and Methods

### 2.1. Microfluidic Chip Design

**Figure 1:**
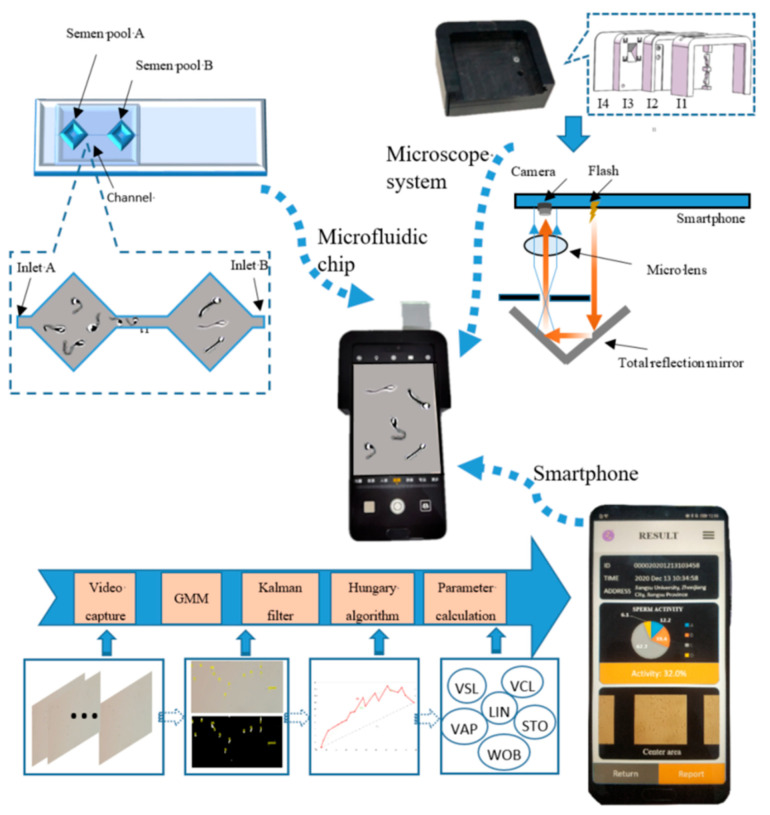
A hand-held platform for boar sperm motility diagnosis based on smartphone, which mainly includes three parts: a microfluidic chip, microscopy system, and smartphone app.

The design of the microfluidic chip is shown in Figure 1. A flow channel substrate made of polydimethylsiloxane (PDMS) printed with a microchannel pattern was adhered to a glass slide. The micro-channel pattern is mainly divided into semen pool A, the channel, and semen pool B. The microfluidic chip has two inlets, A and B, at one end of semen pools A and B, respectively, where the microchannel pattern has a length of 22.7 mm, a width of 7 mm, and a channel width of 2 mm. The depth of the microchannel pattern is designed to be 20 um [22], which can only accommodate one layer of sperm for free plane movement, which is convenient for sperm motility diagnosis.

The microfluidic chip designs two semen pools to simulate the movement of sperm in the fallopian tube. The semen enters the microchannel from inlet A and meets and fuses with the diluent added through inlet B in the center of the channel. Sperm in semen pool A needs to move through the channel to enter semen pool B, just as sperm moves in the fallopian tube and finally combines with the egg to complete the fertilization process.

### 2.2. Microscopic System Design

The schematic diagram of the proposed hand-held platform is shown in Figure 1, which consists of a microscope system, a microfluidic chip, and a smartphone.

As illustrated in Figure 1, the layer structure of the hand-held device consists of layers l1 (width = 84.8 mm, length = 63.4 mm, thickness = 29.4 mm and radius of the round corner = 10 mm), l2 (width = 84.8 mm, length = 63.4 mm and radius of the round corner = 10 mm), l3 (width = 84.8 mm, length = 63.4 mm and radius of the round corner = 10 mm), and l4 (width = 84.8 mm, length = 63.4 mm, thickness = 1 mm and radius of the round corner = 10 mm) from left to right. A notch ((a): width = 24.2 mm, length = 3.5 mm and thickness = 2.2 mm) is included in l1, a lens hole ((b), upper hole: radius = 2.5 mm and depth = 2 mm, lower hole: radius = 1.5 mm and depth = 2.5 m) and a light-passing hole ((c): radius = 2.5 mm and depth = 5.5 mm) are included in l2, and a total reflection prism ((d): width = 12 mm, length = 22 mm and depth = 10 mm) is included in l3. l4 is a bottom layer connected to l1, designed for fixing l2 and l3.

Figure 1 illustrates the composition and position relationship of the parts of the microscope system. The microscope system mainly includes a microlens and a total reflection prism, where a smartphone flash provides the light source for the microscope system, the smartphone camera serves as the eyepiece of the microscope system, and the microlens serves as the objective lens of the microscope system to realize the microscope’s function.

### 2.3. Work Process

The prepared semen sample is added to the microfluidic chip, and the semen flows from the inlet of the microfluidic chip into the semen pool, where the sperm moves in a two-dimensional plane. Next, the microfluidic chip, microscope system, and smartphone are assembled into a hand-held platform. Then, the flash is turned on while turning on the phone camera, and a video of the sperm swimming is recorded after microscopy. Finally, the video is processed by an algorithm on a smartphone app to diagnose the viability of the sperm sample.

### 2.4. Sample Preparation and Data Acquisition

The variety of boar semen used in this article from Large White boars, purchased from China Subu Biotechnology Co., Ltd. (Shanghai, China), and the sperm stock and the diluent are diluted at a ratio of 1:2 and stored in an 18-degree incubator, where the main components of the diluent are sodium chloride, glucose, and sucrose to provide nutrition for the sperm and prolong the life of the sperm; sodium citrate, sodium potassium tartrate, potassium dihydrogen phosphate, etc., are used as buffer substances to balance the pH; egg yolk and milk are used as anti-cold substances; and antibacterial substances such as penicillin, streptomycin, and sulfonamide are used to prevent the reproduction of harmful microorganisms. The pig semen is heated to 37 °C before use, and then the sample is dropped onto the microfluidic chip designed in this paper.

Images are captured using a hand-held system with a Huawei P20 model smartphone. The phone camera is used to adjust to the most appropriate microscopic effect to capture the video. The resolution of the image is 3840 × 2160 pixels, and the frame rate of the video is 29.80 fps. Each pixel in the collected image is 0.074 um.

### 2.5. Sperm-Counting Algorithm

Common target-tracking algorithms are mostly used to detect and track moving targets, while for stationary sperm, they are judged as foreground subjects. Therefore, in this paper, the statistics of moving sperm and stationary sperm are separated; that is, the tracking algorithm is used in determining the activity of moving sperm targets, while the counting algorithm is used in counting the total number of sperm. The design idea of the sperm-counting algorithm is as follows: first, collect the original image; then, grayscale the image; use the OTSU algorithm for threshold segmentation and set the threshold to remove impurities (white blood cells, small particles, etc.); conduct morphological processing (corrosion, swelling) to separate the adhered sperm; and finally, count the connected area, and the result is the total number of sperm.

### 2.6. Sperm Viability Diagnosis Algorithm

#### 2.6.1. Sperm Movement Target Detection

Microscopic videos of boar sperm are captured by the hand-held microscope device. The first step in the dynamic analysis is to obtain the sperm motion trajectory, and the first step in obtaining the motion trajectory is to detect the sperm motion target. The Gaussian mixture model (GMM) [23] is a clustering method that can effectively separate the foreground and moving targets in a video. If the features of the foreground pixels in the video image do not change much over time, it can be assumed that the foreground pixels obey a Gaussian distribution during this time. Suppose there are K unit Gaussian models in the Gaussian mixture model, and each unit Gaussian model describes the motion trend of an image pixel X_t_ with detection time t. The Gaussian distribution of image pixels X_t_ is:

(1) $$p\left(X_{t}\right)=\sum_{i=1}^{k}\omega_{i,t}p_{i}(x)=\sum_{k=1}^{k}\omega_{i,t}\eta (X_{t},\mu_{i,t},\Sigma_{i,t}),$$

where

(2) $$\eta \left(X_{t},\mu_{i,t},\Sigma_{i,t}\right)=\frac{1}{\sqrt{{\left(2\pi \right)}^{n}|\Sigma_{i,t}|}}e^{-\frac{1}{2}{\left(X_{t}-\mu_{i,t}\right)}^{T}{\Sigma_{i,t}}^{-1}(X_{t}-\mu_{i,t})},$$

where $\omega_{i,t}$ denotes the distribution ratio of the i-th Gaussian model at the detection time *t*; $\Sigma_{i,t}$ denotes the covariance of the Gaussian mixture model; and $\eta \left(X_{t},\mu_{i,t},\Sigma_{i,t}\right)$ is the expression of Gaussian probability distribution.

Let *B* be the number of Gaussian distributions in the foreground, and the number of frames is *b*. The expression of *B* is as follows:

(3) $$B=\arg\min(\sum_{K=1}^{b}\omega_{i,t}>\xi ),$$

where argmin ( ) represents the descending order rule and ξ represents the overall prior probability.

#### 2.6.2. Sperm Target Tracking

After detecting the moving sperm target, the sperm target needs to be tracked. The design idea of the tracking algorithm is as follows: use a Kalman filter [24] to predict the state of the next frame based on the existing state, correlate the detected target with the predicted target position, and update the motion trajectory according to the matching result to realize the sperm target tracking process. The data association uses the Hungarian algorithm [25], which is a maximum matching algorithm for finding bipartite graphs, which is a maximum matching algorithm for finding bilateral graphs that uniquely match motion trajectories and detected sperm targets. The process of updating the trajectory set is as follows: Set the threshold to determine whether the target is tracked or lost according to the threshold. If the target is tracked, the track will be updated, and if it is lost, the track will be deleted. If a new target appears, add a new track.

The tracking algorithm can be described as follows:

1. Initialize the Kalman filter;
2. Detect the sperm target in the first frame and record the centroid coordinates;
3. Input the sperm target parameters (position, size, velocity) into the Kalman filter to predict the sperm position in the next frame;
4. Detect the sperm target in the i-th frame and record the centroid coordinates;
5. Use the Hungarian algorithm to associate the predicted sperm location with the detected sperm location, update the trajectory set and Kalman filter;
6. Repeat steps 3, 4, and 5 until the last frame.

Kalman filtering is an algorithm that uses the state equation of a linear system to optimally estimate the system state from the input and output observations of the system. The state equation and observation equation of the Kalman filter system are as follows:

(4) $$x_{k}=Ax_{k-1}+Bu_{k-1}+w_{k-1},$$

(5) $$z_{k}=Hx_{k}+v_{k},$$

In the above Equations (4) and (5), $x_{k}$ is the state of the system at time *k*, and $u_{k}$ is the control quantity of the system at time *k*. *A* and *B* are system parameters, $z_{k}$ is the measured value at time *k*, *H* is the parameter of the measurement system, and $w_{k}$ and $v_{k}$ represent the process and measurement noise, respectively.

The time update equation (the prediction stage) calculates the prior estimate of the state variable and the prior estimate of the error covariance according to the state estimate at the previous moment. The process is as follows:

(6) $$K_{k}=P_{k}^{-}H^{T}{\left(HP_{k}^{-}H^{T}+R\right)}^{-1},$$

(7) $${\hat{x}}_{k}={\hat{x}}_{k}^{-}+K_{k}\left(y_{k}-H{\hat{x}}_{k}^{-}\right),$$

(8) $$P_{k}=(I-K_{k}H)P_{k}^{-},$$

### 2.7. Calculation of Sperm Motility Parameters

Dynamic analysis is conducted on the motion curve of each sperm. A video duration *T* is assumed. The velocity of curve line (VCL), velocity of straight line (VSL), velocity of average path (VAP), linearity (LIN), wobble (WOB), and straight (STR) are given by Equations (9) to (14). *L* is the path of the curve line. $x_{t}$ indicates the position of the sperm at time t. *S* means the average path of the trajectory.

(9) $$VCL=\frac{L}{T},$$

(10) $$VSL=\frac{x_{t=T}-x_{t=0}}{T},$$

(11) $$VAP=\frac{S}{T},$$

(12) $$LIN=\frac{VSL}{VCL},$$

(13) $$WOB=\frac{VAP}{VCL},$$

(14) $$STR=\frac{VSL}{VAP},$$

## 3. Results and Discussion

### 3.1. Sperm Image Video Collection

**Figure 2:**
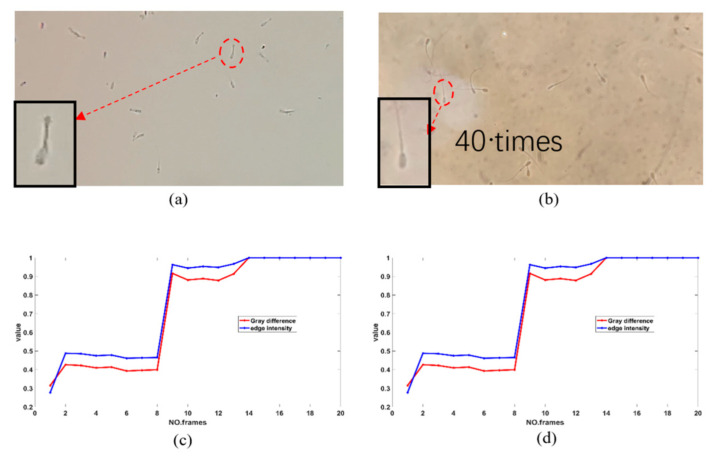
(**a**) The sperm image in the hand-held system in this paper. (**b**) The sperm image under the 40× objective lens of a Nikon microscope. (**c**) The sharpness evaluation result of the image sequence in the video captured by the hand-held system. (**d**) The sharpness evaluation result of the image sequence in the video captured by the Nikon microscope.

A hand-held system was used to acquire images of spermatozoa for comparison with a conventional optical microscope. Figure 2a shows an image of boar sperm collected by the hand-held system in this paper, and Figure 2b shows an image of the boar sperm collected under the 40± objective lens of a Nikon microscope. It can be seen that although some of the tail information is lost, the sperm head information and most of the tail information is preserved. The magnification of the hand-held system is about 38×, which is close to the image acquisition of a Nikon microscope with a 40× objective lens. The captured images can be used for sperm identification and tracking.

The sharpness of the image sequences in the video captured by the hand-held system is evaluated and compared with the sharpness of the image sequences in the video captured by the Zeiss Axiocam 506 microscope camera. The SDF (defined evaluation function based on SMD (sum of greyscale difference modulus)) and TDF (defined evaluation function based on Tenengrad) [26,27] were used, with the following equations:

(15) $$G=│f\left(x,y\right)-f(x+1,y)│+│f\left(x,y\right)-f(x,y+1)│,$$

(16) $$SDF=\frac{1}{M\times N}(\sum │I\left(x,y\right)-I(x,y-1)│+\sum │I\left(x,y\right)-I(x+1,y)│,$$

where *I*(*x*,*y*) is the grayscale value of the point and *M* × *N* is the total number of image pixels.

(17) $$S\left(x,y\right)=\sqrt{G_{x}\times I\left(x,y\right)+G_{y}\times I(x,y)},$$

(18) $$TDF=\frac{1}{M\times N}\underset{M}{\sum }\underset{N}{\sum }{S(x,y)}^{2},$$

where *G_x_*, *G_y_* are Sobel operators and S(*x*,*y*) is the gradient value of the point (*x*,*y*) on the image.

Figure 2c,d show the normalized results of the definition evaluation of 50 frames of images extracted from the videos captured by the hand-held system and the Zeiss Axiocam 506 microscope camera, respectively. The sharpness evaluation of the images in the video taken by the hand-held system fluctuates around 0.9. In contrast, the image sequences captured by the Zeiss Axiocam 506 microscope camera performed slightly better.

### 3.2. Sperm Image Analysis and Quantity Statistics

**Figure 3:**
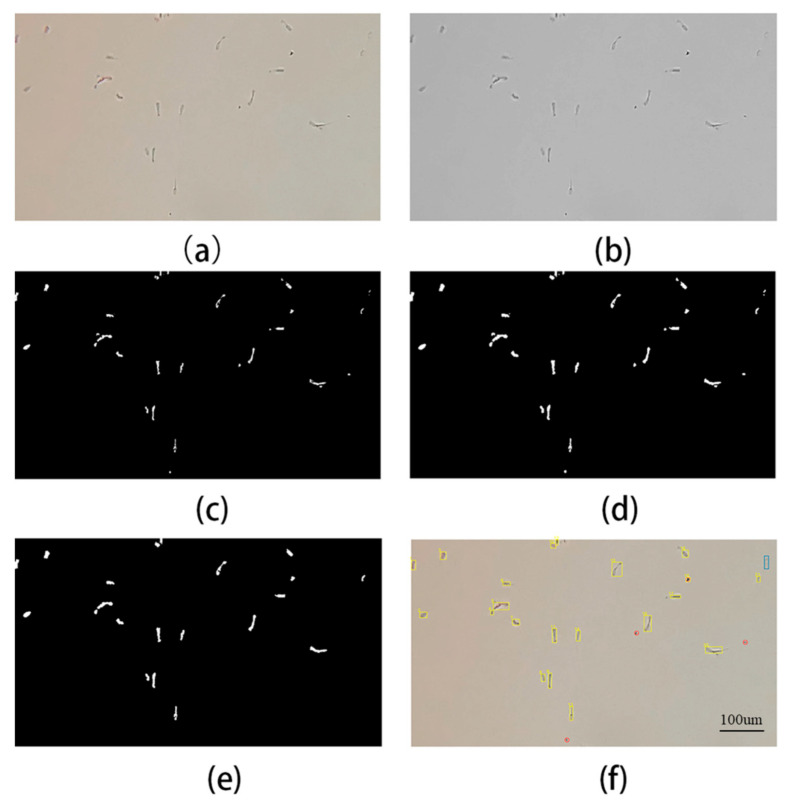
(**a**) The original image captured by the camera. (**b**) The image after grayscale. (**c**) The image after threshold segmentation using the OTSU algorithm. (**d**) The image after morphological operation. (**e**) The image after removing impurities. (**f**) The recognition result image.

After obtaining the sperm video, in order to count the number of sperm, the video image needs to be pre-processed first. Figure 3a shows the original image captured by the camera; Figure 3b is the image after grayscale has been applied; Figure 3c is the image after threshold segmentation using the OTSU algorithm with a threshold of 0.6235; Figure 3d is the image after morphological operations, filling the holes, and performing expansion and erosion operations have been performed; Figure 3e is the image after removing impurities and setting the threshold (70 pixels) to remove small particles of impurities (white blood cells, dust) so that a binary image with only sperm left is obtained; and Figure 3f is the recognition result image, where the yellow rectangle represents the recognized sperm target, the red circle represents the impurities (white blood cells, dust), and the blue rectangle represents the unrecognized sperm target. When the red circle overlaps with the yellow rectangle, it means an incorrect recognition.

**Figure 4:**
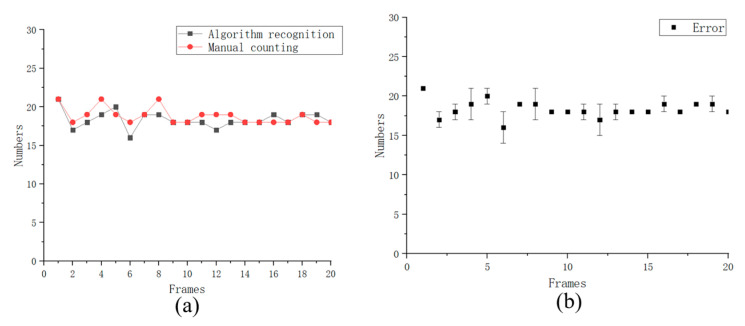
(**a**) Comparison of the results of counting using the algorithm proposed in this paper with manual counting. (**b**) The error diagram.

The 20-frame image sequence in the video was intercepted, which is further identified and counted with a certain algorithm. The result is then compared with manual calculation, as shown in Figure 4. The calculation result of the algorithm is basically consistent with the manual calculation result, and the error rate is less than 5%. Thus, the total recognition rate is 96.08%. The accuracy is much higher than the 91.77% accurate sperm head detection method proposed by Valiuškaitė et al. in 2020 with a deep learning method based on region-based convolutional neural networks (R-CNNs) [28]. And we have also improved the detection accuracy compared with the work conducted by Pan et al. in 2022, which was 94% [29].

### 3.3. Sperm Trajectory Tracking

As the sperm was identified, its centroid coordinates were saved for developing a tracking algorithm. Three videos with 90 frames in three seconds were captured and used for further tracking. The results shown in Table 1 count the frames in which each sperm appeared and disappeared, as well as the tracking rate, which was calculated as follows:

(19) $$R_{t}=\frac{F_{t}}{F_{w}}$$

where *R_t_* represents the tracking rate, *F_t_* represents the total number of frames tracked by a certain sperm *ID*, and *F_w_* represents the total number of frames that a certain sperm appears during the entire video sequence.

The data provided in the table were further calculated and summarized. Sperms with a 100% tracking rate accounted for 70.59% of the total. Sperms with a tracking rate less than 50% accounted for 5.88%. The average tracking rate was 86% on the whole.

**Figure 5:**
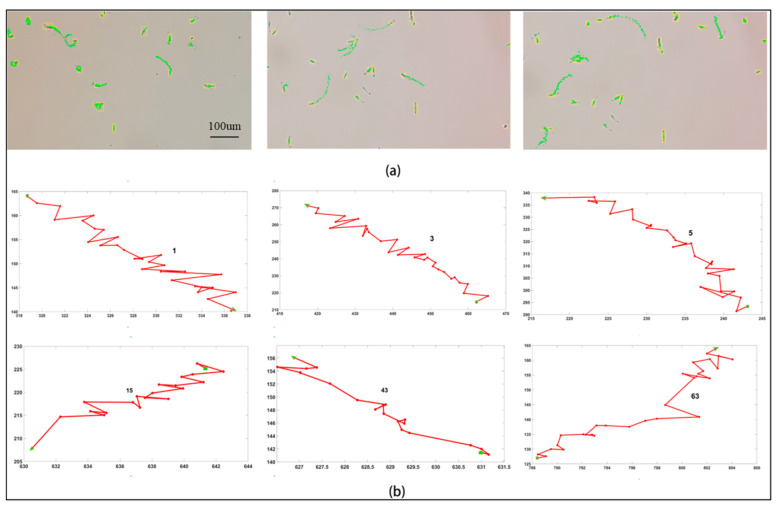
(**a**) Sperm trails in three videos. (**b**) Some of the individual sperm trajectories: the starting point is the green rectangle and the ending point is the green arrow. It should be noted that the speed of each sperm is different, and the coordinate axis should be considered (the X- and Y-axis units are in pixels).

The target sperm was tracked, and the centroid of each frame was recorded. The movement of sperm is described with the curve obtained by connecting the centroids of the trajectories. Figure 5 shows part of the sperm trajectory tracked in the video sequence, in which Figure 5a shows the total sperm motion trajectory in the three videos, and Figure 5b shows some of the individual sperm motion trajectories. As we can see, the green rectangle represents the starting point of the sperm, and the green arrow represents the endpoint of the sperm. The sperm could move in a free plane on the proposed microfluidic chip. Also, the sperm has a general tendency to move forward in a zigzag process and a random direction.

### 3.4. Sperm Motility Parameters Analysis

After getting the sperm trajectory, the above algorithm can be used to find the sperm motility parameters (*VCL*, *VSL*, *VAP*, *LIN*, *STR*, *WOB*). After obtaining the sperm trajectory, the sperm motility parameters (*VCL*, *VSL*, *VAP*, *LIN*, *STR*, *WOB*) can be obtained using the algorithm in II. G. And according to the classification criteria of the World Health Organization, sperm is divided into four grades: A (fast progressive movement), B (slow progressive movement), C (non-progressive movement), and D (immobility). Some of the sperm motility parameters are shown in Table 2.

### 3.5. Microfluidic Chip Simulation and Verification

**Figure 6:**
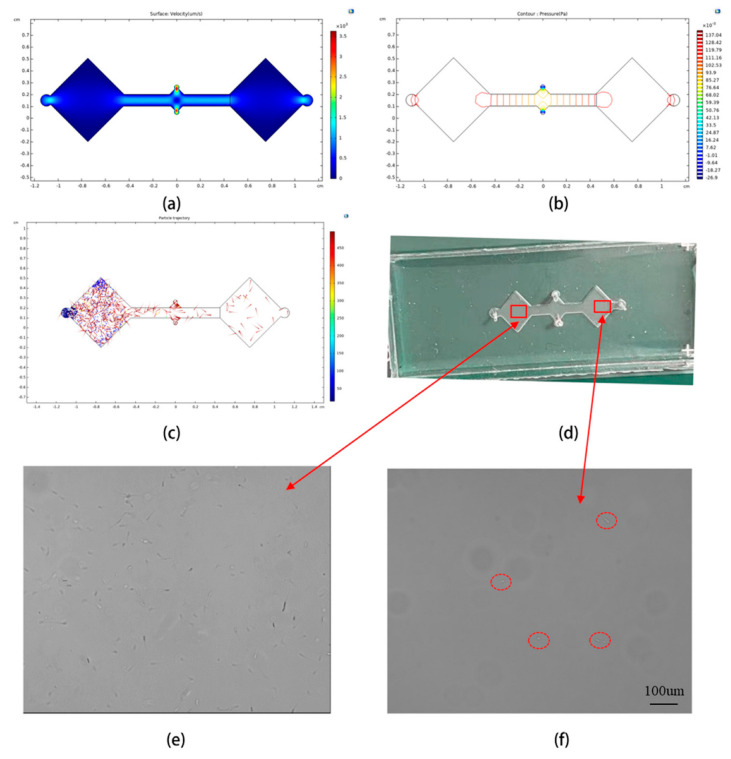
(**a**–**c**) The simulation results of the microchannel in comsol. (**a**) The fluid surface velocity distribution. (**b**) The pressure contour distribution. (**c**) The particle motion trajectory distribution of three different speeds. (**d**) Microfluidic chip. (**e**,**f**) The experimental results of the microfluidic chip simulating the movement of sperm in the fallopian tube, which are the microscopic images of semen pools A and B, respectively.

Comsol multiphysics 5.3a software was used to simulate the movement of sperm in the oviduct for fertilization on a designed microfluidic chip. The semen and diluent are set up to enter the microfluidic chip channel from both inlet A and inlet B. The fluid density is 0.998 mol/m^3, the dynamic viscosity is 0.00089 Pa*s, and the initial velocity is 5 mm/s. At the same time, three kinds of particles (velocities of 50 um/s, 10 um/s, 0 um/s) are released from entrance A to simulate three different sperm velocities. In the simulation process, the sperm was assumed to be spherical, with a diameter of 8 um, and the real sperm head length is about 8 um, and the tail length is around 40 um. The simulation results are shown in Figure 6a–c. Figure 6a is the velocity distribution of the fluid on the surface of the microfluidic chip, Figure 6b is the pressure contour distribution of the microfluidic chip, and Figure 6c is the distribution of three kinds of sperm trajectories in the microfluidic chip. A total of 1200 particles are released from entrance A. Each particle is released 20 times at a time, with an interval of 0.5 s each time. After 200 s, a total of 30 particles are counted entering the semen pool B, and the transmission probability is 2.5%.

The microfluidic chip is designed by AutoCAD 2019, made into a film according to the micro-pattern, and pasted on a 20 µm thick photosensitive dry film, which in turn was pasted on a copper plate. After exposure, the photosensitive dry film was put into the developing solution for developers to form the fluidic channel die micropattern. The flow channel substrate is composed of PDMS (Dow Corning), and approximately 5 g PDMS base and curing agents are mixed at a ratio of 10:1. The mixed material was then poured into the acrylic sheet mold with a rectangle cavity. Air bubbles were removed via degassing. The substrate was baked at 60 °C for one hour. The air inlet and outlet of the microfluidic device were punched with needles or punches of the same size as the connecting pipe. The PDMS surface was treated with oxygen plasma for 30 s and bonded to the glass slide to prevent liquid leakage.

The fabricated microfluidic chip is shown in Figure 6d. A BD Ultra-Fine Needle Insulin Syringe (U40) was used to inject the sample into the microfluidic chip. One syringe controls the injection of semen into inlet A, and the other syringe controls the injection of diluent into inlet B. After the two liquids meet at the center of the microfluidic chip and fill the entire channel, the microfluidic chip was placed into the microscopic device described in this article for observation. The results are shown in Figure 6e,f. Figure 6e is the microscopic image of semen pool A, where it can be seen that the sperm concentration is very high, while in the microscopic image of semen pool B (Figure 6f), the number of sperm is very small, which confirms the simulation results in Figure 6c.

### 3.6. Sperm Motility Parameters Analysis

**Figure 7:**
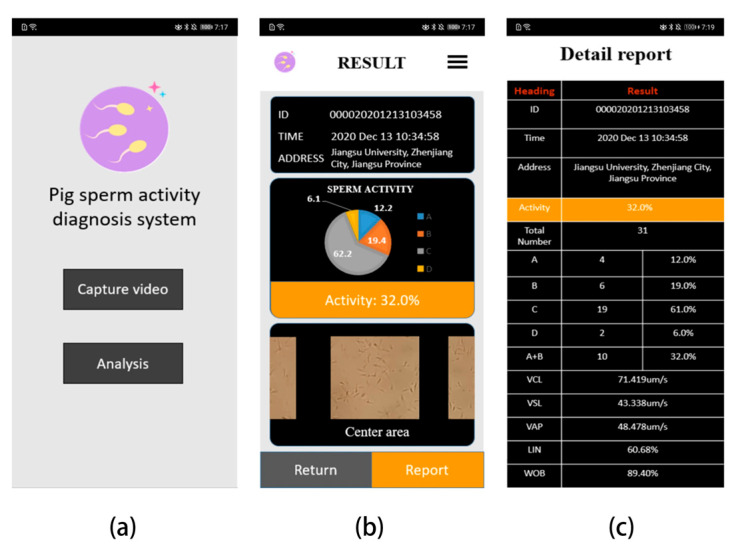
Screenshots of the developed app interface. (**a**) Main program interface, provides recording video buttons and analysis buttons. (**b**) The result display interface after the analysis is completed. (**c**) The detailed report description interface.

The smartphone application is developed using Google’s android studio, the interface is designed using the XML language, the program is developed in the Java language, and the image processing part uses the OpenCV library. Figure 7a shows the main interface. There are two buttons: one is the button for shooting video, the other is the analysis button. Figure 7b is the result display interface, which provides a pie chart of the percentage of sperm activity classification, and a report button. Clicking this button jumps to the page shown in Figure 7c to view the detailed report; in Figure 7c, there is the detailed sperm number distribution of A, B, C, and D, as well as the statistics of the sperm motility parameters.

## 4. Conclusions

In this article, a hand-held platform based on smartphones was developed to diagnose sperm motility. The microscopic effect of the developed hand-held platform is equivalent to that of a microscope’s 40× objective lens, and the captured video is clear and stable. A smart diagnostic application was developed for identifying and tracking sperm targets. The detection algorithm used has a recognition rate of 96.08%, and the tracking algorithm has an average tracking rate of 86%. It can detect sperm and determine their motility for classification. In addition, the designed microfluidic chip channel has a thickness of 20 um, which can only accommodate one layer of sperm for free-plane motion, avoiding the stacking of sperm. Two semen pools and one channel were designed to simulate the movement of sperm in the oviduct. In conclusion, we developed a smartphone-based hand-held platform and a smart diagnostic app for sperm motility testing during boar reproduction. It is easy to operate and portable compared to traditional microscope testing.

## Figures and Tables

**Table 1:** Sperm tracking by ID.

ID	Appear Frame	Disappear Frame	*F_t_* (Frames)	*F_w_* (Frames)	*R_t_* (%)
1	1	72	72	72	100
2	1	55	55	90	61.11
3	1	44	45	45	100
5	1	90	90	90	100
9	5	31	27	42	64.29
15	6	90	85	85	100
17	8	52	45	83	54.22
24	9	29	11	62	17.74
25	12	90	79	79	100
27	13	90	78	78	100
43	21	90	70	70	100
45	23	66	44	68	64.70
56	25	61	37	37	100
63	25	90	66	66	100
74	28	90	63	63	100
87	28	90	63	63	100
89	54	90	36	36	100

**Table 2:** Movement parameters and activity of sperm.

ID	*VCL* (um s^−1^)	*VSL* (um s^−1^)	*VAP* (um s^−1^)	*LIN* (%)	*STR* (%)	*WOB* (%)	*GRADE*
1	13.63	6.02	8.63	44.17	69.76	63.32	C
3	27.42	14.65	22.57	53.13	64.91	82.31	B
5	19.44	10.51	13.92	54.06	75.50	71.60	C
15	11.95	6.27	8.17	52.47	68.37	76.74	*C*
25	15.35	4.99	7.14	32.51	69.89	46.51	*C*
27	10.19	3.63	5.94	35.62	58.29	61.11	*C*
43	5.86	4.62	5.35	78.83	94.37	86.36	*D*
56	20.65	12.46	17.79	60.34	70.04	86.15	*C*
63	13.11	8.05	9.93	61.40	81.07	75.74	*C*
89	25.39	10.66	16.28	39.39	65.48	64.12	*B*

## Data Availability

Data are all contained within the article.
